# Causal association between dried fruit intake and risk of osteoarthritis: A Mendelian randomization study

**DOI:** 10.1097/MD.0000000000037710

**Published:** 2024-04-05

**Authors:** Ruiming Liang, Weixing Zhong, Shuaidi Ze, Yuxiang Qiao, Lixia Yuan

**Affiliations:** a School of Traditional Chinese Medicine, Southern Medical University, Guangzhou, China.

**Keywords:** causal relationship, dried fruit intake, genome-wide association study, Mendelian randomization, osteoarthritis, single nucleotide polymorphism

## Abstract

This study aimed to examine whether dried fruit intake is causally associated with Osteoarthritis (OA). A two-sample Mendelian randomization (MR) analysis using the inverse-variance weighted (IVW), weighted median (WM), and MR-Egger regression methods was performed. We used the publicly available summary statistics data sets of genome-wide association studies (GWAS) meta-analyses for dried fruit intake in individuals included in the UK Biobank (n = 421,764; MRC-IEU consortium) as the exposure and a GWAS publicly available in PubMed for OA (total n = 484,598; case = 39,515, control = 445,083) as the outcome. We selected 41 single nucleotide polymorphisms at genome-wide significance from GWASs on dried fruit intake as the instrumental variables. The IVW method showed evidence to support a causal association between dried fruit intake and OA (beta = −0.020, SE = 0.009, *P* = .039). MR-Egger regression indicated no directional pleiotropy (intercept = 1E-05; *P* = .984), but it showed no causal association between dried fruit intake and OA (beta = −0.020, SE = 0.043, *P* = .610). However, the WM approach yielded evidence of a causal association between dried fruit intake and OA (beta = −0.026, SE = 0.012, *P* = .026). Cochran’s Q test showed the existence of heterogeneity, but the statistics of *I*^2^ showed low heterogeneity. The results of MR analysis support that dried fruit intake may be causally associated with a decreased risk of OA.

## 1. Introduction

Osteoarthritis (OA) is the most common joint disease, estimated to affect more than 240 million people worldwide. OA is adults’ most common cause of limited activity.^[1]^ The macronutrients, micronutrients, and other health-promoting bioactive compounds in nuts and dried fruits may cooperate through various mechanisms to regulate cardiometabolic and additional non-communicable disease risks. Experimental studies, prospective studies, human clinical trials have reported beneficial effects of nut consumption on various health outcomes.^[2]^ In recent years, clinical and epidemiological studies have shown the protective effect of fruits and their polyphenols on arthritis.^[3,4]^

Mendelian randomization (MR) is a technique that uses genetic variation as an Instrumental variable (IV) to assess whether the observed association between risk factors and outcome is consistent with a causal effect.^[5]^Two-sample MR estimates the causal impact of data on contacts and outcomes in different samples.^[6]^Recent MR studies support a causal relationship between OA and lifestyle-related obesity, coffee intake, Bdensity, sleep disturbance, decreased serum calcium and LDL cholesterol levels.^[7]^ No studies have reported using MR methods to test the causal effect of dried fruit intake and OA risk. Therefore, this study used a two-sample MR analysis to investigate whether dried fruit intake is causally associated with the risk of developing OA.

## 2. Materials and methods

### 2.1. Data sources and selection of genetic variation

**Figure 1.**
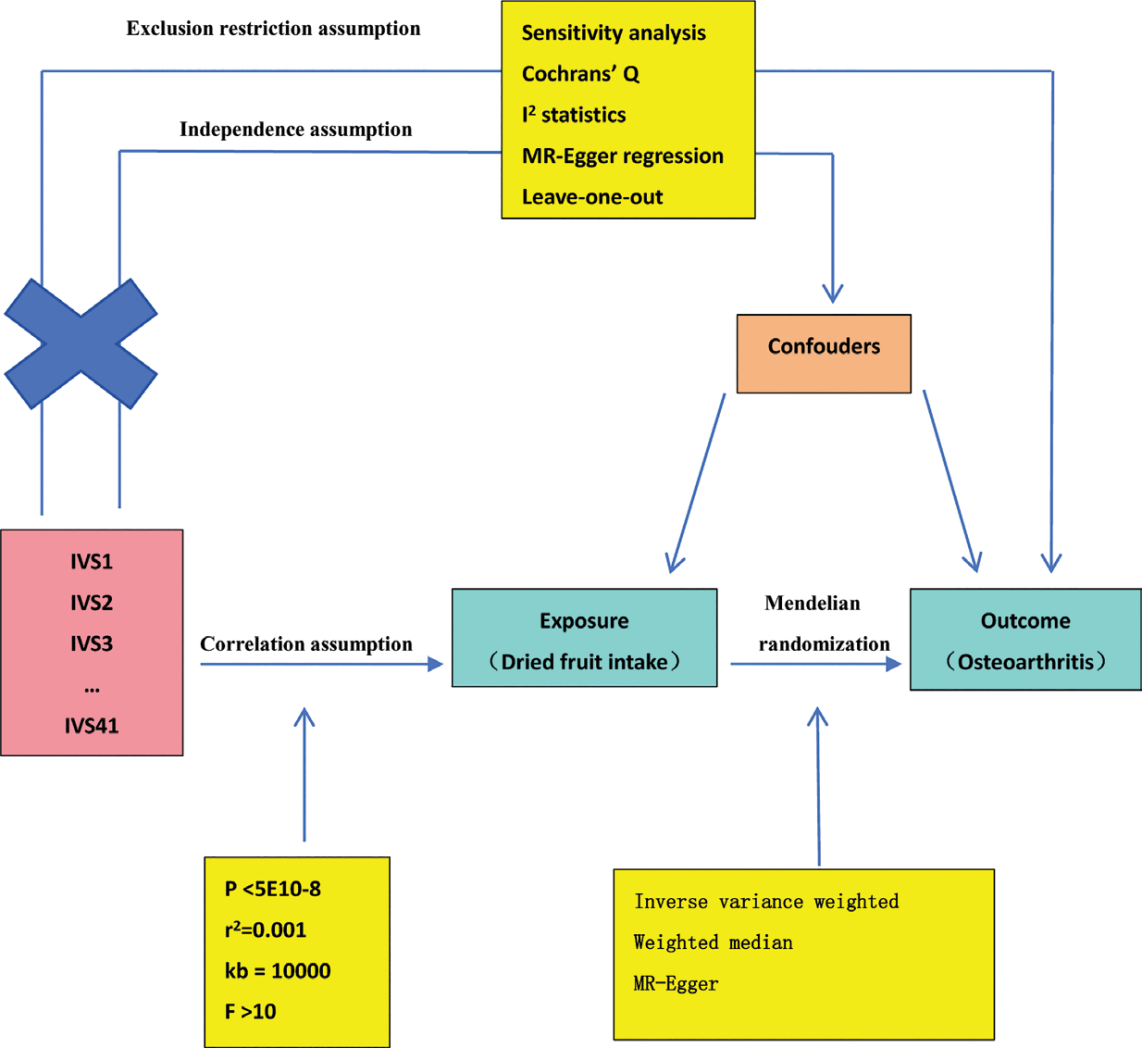
Schematic diagram of the study design in this two-sample MR analysis. Forty-one important IVS for dried fruit intake and osteoarthritis were selected and then explored for bidirectional causality. The 3 basic assumptions of MR analysis, correlation assumption, independence assumption, and exclusion restriction assumption, are illustrated in this schematic diagram. IVS = instrumental variables, MR = mendelian randomization.

A Schematic diagram of the study design in this two-sample MR analysis is outlined in Figure 1. Single nucleotide polymorphism sites (SNP) published by the MRBase database were used as an IV to determine the effect of risk between dried fruit intake and OA. The website collects a large number of summary statistics from hundreds of genome-wide association studies (GWAS). We used the publicly available summary statistics data sets of GWAS meta-analyses for dried fruit intake in individuals included in the UK Biobank (id:ukb-b-16576; n = 421764; MRC-IEU consortium) as the exposure^[8]^ and a GWAS publicly available in PubMed for OA (id: ebi-a-GCST90038686; total n = 484598; case = 39515, control = 445083) as the outcome^[9]^ (Table 1). This study was based on publicly available data, so no additional ethical approval or consent was required.

**Table 1:** Summary of genome-wide association study data in this MR study.

Exposure/outcome	Web source (http://www.mrbase.org/)	GWAS ID	Sample size	SNP size	First author	Consortium	Year	Population studied
Dried fruit intake	1319: Output from GWAS pipeline using Phesant derived variables from UKBiobank	ukb-b-16576	421764	9851867	Ben Elsworth	MRC-IEU	2018	European
Osteoarthritis	https://pubmed.ncbi.nlm.nih.gov/33959723/	ebi-a-GCST90038686	484598	9587836	Dnerta HM	NA	2021	NA

GWAS = genome-wide association study, MR = mendelian randomization, SNP = single nucleotide polymorphism.

### 2.2. Selection of IVs

MR analysis has three core assumptions, namely correlation, independence, and exclusion restriction.^[10]^ It calls for genetic variation associated with exposure but is not a potential confounder for exposure. First, genome-wide divergent SNPs (*P* < 5E10-8) and their linkage disequilibrium (*r*^2^ = 0.001, kb = 10,000) were selected as the IVs and then eliminated in linkage disequilibrium to ensure that the IVs satisfied independence. Genetic variation is not associated with confounders. Genetic variation was not associated with the outcome variables. The *F* statistic was used to assess the statistical strength of the correlation between SNPs and exposure. The *F* statistic is (*F *= *R*^2^/(1 − *R*^2^) · (*N*−*K*−*1*)/*(K*−1)), where *N* is the sample size of the dried fruit intake database, *K* is the number of SNPs, *R*^2^ represents the proportion of dried fruit intake explained by SNPs, calculating the formula *R*^2^ = 2·(1 − *EAF*)·*EAF* · β^2^. EAF is the effect allele frequency (effect allele frequency, EAF) for each SNP, and β is the effect value for the allele.^[11,12]^ A threshold of *F* < 10 was used to define a weak IV, considered as a weak association between SNPs and exposure.^[13]^

### 2.3. Statistical analysis of MR

A two-sample MR analysis used summary statistics from different GWAS to estimate the causal effect of exposure (dried fruit intake) on the outcome (OA). The 41 SNPs from the GWASs data for dried fruit intake and OA were used as IV (Table 2) to assess the causal relationship between dried fruit intake and OA risk. The inverse variance weighted analysis method (IVW)^[13–15]^ was used as the primary analysis method, with the weighted median (WM)^[16]^ and MR-Egger regression^[17]^ as the auxiliary methods. IVW combined the Wald estimates for each SNPs to obtain the overall estimate.^[18]^ Tests were considered statistically significant at *P* less than .05. All MR analyses were on the MR Base platform (application version: 1.4.3 8 a 77 eb [October 25, 2020], R version: 4.0.3).^[19]^

**Table 2:** MR estimates from each method of assessing the causal effect of dried fruit intake on the risk of osteoarthritis.

MR method	nSNP	Beta	SE	OR	95% CI	Association *P* value	Cochran Q statistic	*I * ^2^	Heterogeneity *P* value
MR Egger	41	−0.020	0.043	0.980	0.900–1.066	.640	57.81	0.325	.027
Weighted median	41	−0.026	0.012	0.974	0.951–0.997	.025	–	–	–−
Inverse variance weighted	41	−0.020	0.009	0.981	0.963–0.999	.039	57.81	0.309	.034

*I*^2^ = (Q − df)/Q.

Beta = beta coefficient, nSNP = number of single nucleotide polymorphism, SE = standard error.

The analysis results were presented as odds ratios (OR) and 95% confidence intervals (95% CI). OR > 1 is a positive correlation, indicating that exposure factors are adverse outcomes; OR < 1 is a negative correlation, meaning that exposure factor is a favorable factor for outcome.

### 2.4. Sensitivity analysis

The sensitivity analysis included the heterogeneity test, the horizontal pleiotropic test, and the Leave-one-out sensitivity test. Heterogeneity is the variability of the causal estimates obtained for each SNP (i.e., the consistency of the causal estimates across all SNP). We used the MR-Egger method and IVW statistical methods to record Q values and degree-of-freedom Q-Q-df and *P* values. *P* > .05 in Cochran’s Q heterogeneity test showed no heterogeneity.^[20]^

The *I*^2^ statistic is the proportion of the observed inter-study variation (due to true heterogeneity rather than that observed by chance). The formula was calculated as *I*^2^ = 100% *(Q – df)/Q. Q is Cochran’s Q heterogeneity statistic and df is the degree of freedom. Since 0 is taken when *I*^2^ is negative, the value of *I*^2^ is between 0% and 100%. An *I*^2^ of 0% indicates no heterogeneity, and larger values indicate more significant heterogeneity. *I*^2^ > 56% reveals great heterogeneity among studies; if *I*^2^ < 31%, they can be considered for *I*^2^ between 31% and 56%. Low heterogeneity suggests an increased reliability of MR analysis.^[21,22]^

Multiple instrumental variables were tested for horizontal pleiotropy by the MR-Egger intercept test, and the robustness of the results was assessed. The intercept represents the average pleiotropic effect of the genetic variant (the average direct effect and result of the variation). Non-zero intercept (MR-Egger test) suggests directional pleiotropy. Set *P* > .05, meaning no pleiotropy. If the IV affects the outcome through factors other than exposure factors, it indicates that the IV is pleiotropic, and the assumption of independence and exclusivity is not valid.^[23]^

Sensitivity analysis was performed using a Leave-one-out sensitivity test. It mainly calculates the MR results of the remaining IV after eliminating the IV one by one. If the MR results and the total results estimated by the other IV after excluding one IV differ significantly, it indicates that the MR results are sensitive to the IV.

## 3. Results

### 3.1. Studies included in the meta-analysis

#### 3.1.1. IVs for MR.

**Figure 2.**
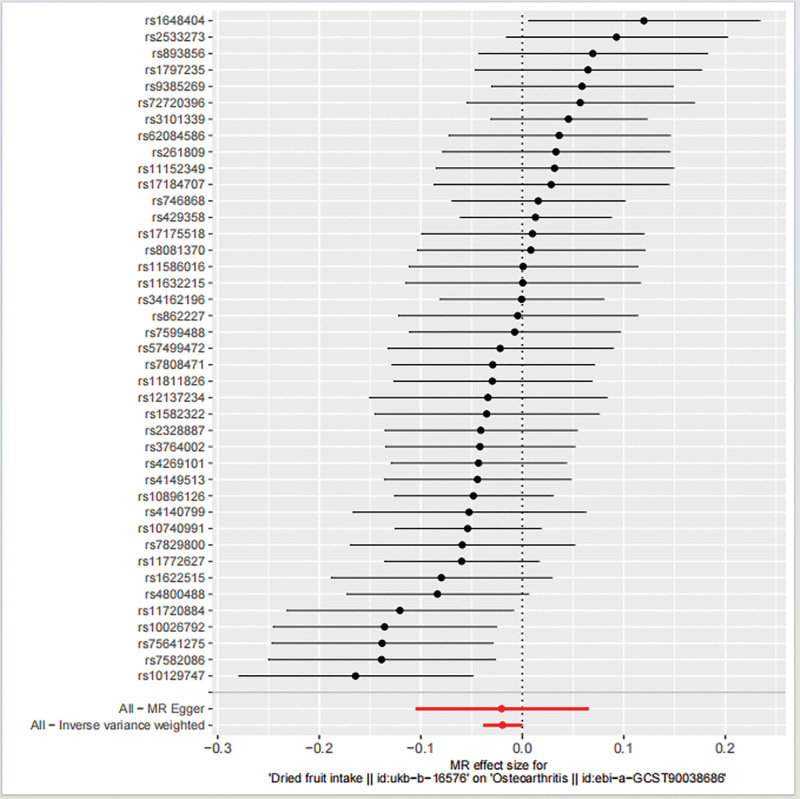
Forest plot of the causal effects of single nucleotide polymorphisms associated with dried fruit intake on osteoarthritis. Red line represents the MR results of the MR-Egger test and the IVW method. IVW = inverse variance weighted, MR = mendelian randomization.

Forty-one independent SNPs from GWASs of dried fruit ingestion were selected as IV (The MRkeep of rs11037497 and rs7916868 shows False because of the palindrome structure). They were all genome-wide associated with dried fruit intake (Table 2; Fig. 2). The distribution of the *F* statistic corresponding to calculating single SNPs has a range of 29.7–81.2, and all *F* values > 10. therefore, the weak IV bias is negligible.

#### 3.1.2. MR results.

**Figure 3.**
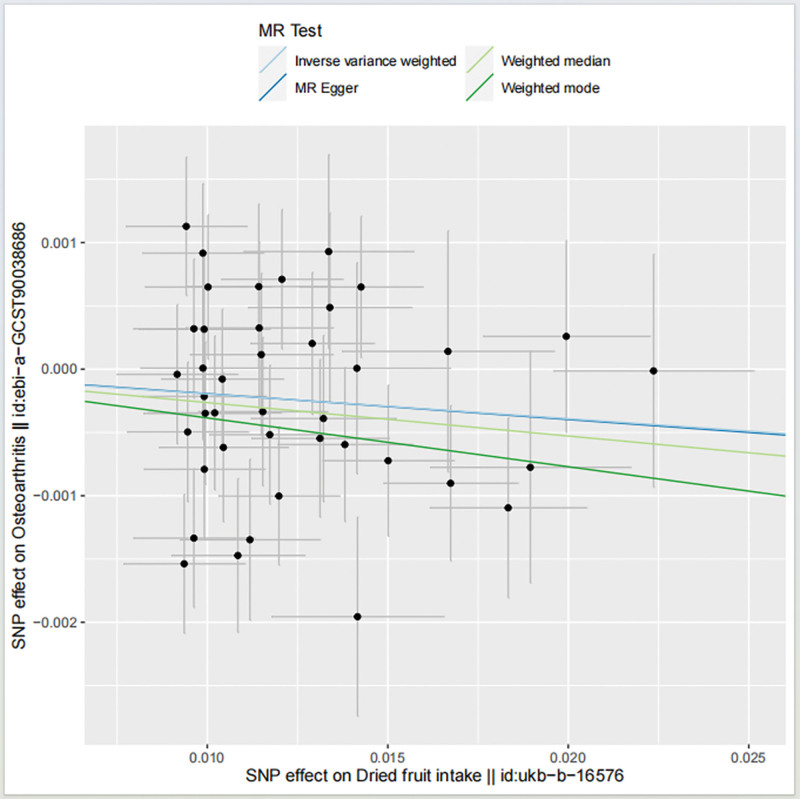
Scatter plots of genetic associations with dried fruit intake against the genetic associations with osteoarthritis. The scatterplot depicts the causal relationship between dried fruit intake and osteoarthritis. The slopes of each line represent the causal association for each method. The blue line represents the inverse-variance weighted estimate, the green line represents the weighted median estimate, and the dark blue line represents the Mendelian randomization-Egger estimate. Each dot represents an IV, vertical and horizontal lines in the center of the dot represent the 95% CI. CI = confidence interval, IV = instrumental variable.

The IVW method showed evidence to support a causal relationship between dried fruit intake and OA (beta = −0.020, SE = 0.009, *P* = .039; Table 2; Figs. 2 and 3). The MR-Egger analysis showed no causal relationship between dried fruit intake and OA (beta = −0.020, SE = 0.043, *P* = .610; Table 2; Figs. 2 and 3). However, the WM method showed a causal relationship between dried fruit intake and OA (beta = −0.026, SE = 0.012, *P* = .026; Table 2; Fig. 3). The correlation between dried fruit intake and OA was inconsistent between the MR Egger and the WM methods. The IVW and WM methods suggested a causal relationship between dried fruit intake and OA risk, while the MR-Egger method suggested no causal relationship. Given the advantage of WM estimates with higher estimation accuracy than the MR-Egger analysis, the results of the MR analysis may support a potential causal relationship between dried fruit intake and OA.

Using the IVW method, dried fruit intake and OA risk were stable [OR = 0.980630056, 95% CI (0.96258306–0.999015405)], and OA risk [OR = 0.973935694, 95% CI (0.951445188–0.996957837)]. Suggesting a negative causal association between dried fruit intake and OA. However, the MR-Egger method indicates that the relationship is unstable [OR = 0.97981647, 95% CI (0.900268704–1.066393079)] (Table 2).

### 3.2. Sensitivity analysis

In Cochran’s Q heterogeneity test (MR Egger *P* = .02664; IWW *P* = .03388) (Table 2), however, our *I*^2^ statistic showed low heterogeneity (IVW *I*^2^ = 0.308078187) (Table 2).

**Figure 4.**
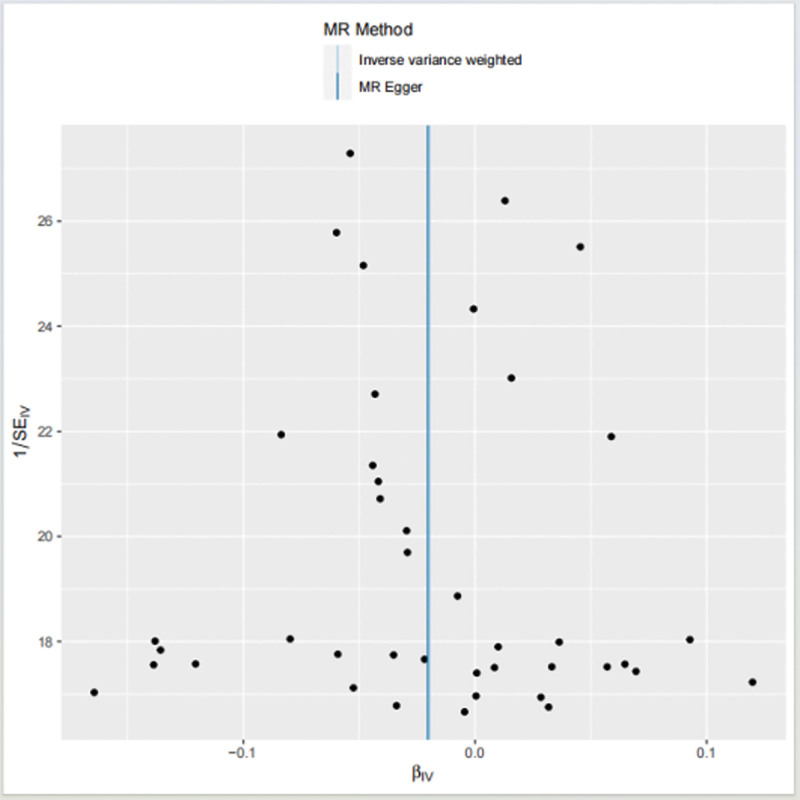
Funnel plot to assess heterogeneity. The blue line represents the inverse variance weighted estimate, and the dark blue line represents the Mendelian randomization-Egger estimate.

MR-Egger regression showed horizontal pleiotropy is unlikely to affect the results (intercept = 1E–05; *P* = .984) and showed that the instrumental variables do not significantly affect the outcome through pathways other than exposure. The asymmetry in the funnel plot indicates directional level pleiotropy, which may bias the MR method; however, the funnel plot shows evidence of better symmetry (Fig. 4), indicating increased reliability of MR analysis.

The Leave-one-out sensitivity test analysis results indicated that no single SNP affected the IVW site assessment.

## 4. Discussion

Studies have shown that nutritional interventions provide health benefits for preventing and managing knee or hip OA through weight loss, reduced inflammation, and antioxidant capacity. However, because data are limited to mixed results, high-quality evidence, including longitudinal studies and clinical trials, is needed to understand whether nutritional supplementation effectively prevents or manages OA.^[24]^ The prevalence of OA was lower among participants adhering to the Mediterranean diet.^[25]^ However, whether dried fruit intake is causally related to OA remains unclear. This study used three estimation methods (IVW, WM, and MR-Egger regression). Our study suggests a possible causal relationship between dried fruit intake and OA. Although MR estimates using IVW, MR Egger, and WM analyses were inconsistent, IVW and WM analyses supported a causal relationship between dried fruit intake and OA. Considering that compared to the MR-Egger analysis, the WM estimator has the advantage of retaining greater precision in the estimates,^[16]^ this MR analysis indicates the potential causal role of dried fruit intake in OA risk, which also provides evidence support for dietary intervention to strengthen the prevention and management of OA.

However, the biological mechanisms of genetic variation as an IV could be more precise, considering that dried fruit intake may influence the occurrence and development of OA through the following mechanisms. First, the pathological progression of OA is closely related to oxidative stress. However, dried fruits contain many bioactive compounds, including phenols, flavonoids, carotenoids, proanthocyanidins, stilbenes, chalcone/dihydrochalcone, and phytoestrogens. These natural antioxidants can alleviate oxidative stress and chondrocyte damage and thus play a role in the management of OA.^[26]^ Ellagic acid (EA) is a natural polyphenol extracted from fruits or nuts, which can effectively inhibit oxidative stress induced by IL-1 and improve chondrocyte oxidative stress-induced dysfunction; significantly inhibit apoptosis and aging, and reduce the expression of pro-inflammatory factors and metalloproteinases; and through up-regulating Keap1/Nrf2 signaling pathway, weaken oxidative stress and play a protective effect on chondrocytes.^[27,28]^ Limonin reduces IL-1 levels of β-induced pro-inflammatory cytokines and reduces the biosynthesis of IL-1*β*-stimulated chondrogenic catabolic enzymes in chondrocytes to reduce inflammation, and exerts a protective effect on OA through the Nrf2/HO-1/NF-*κB* signaling pathway.^[29]^ Sinensetin (SIN) can reduce the expression of inflammatory factors and ECM breakdown factors, delay the destruction of articular cartilage and regulate the NF-κB/NLRP3 signal pathway by regulating the expression of articular cartilage SERPINA3, inhibit the apoptosis of chondrocytes in rat knee joint, and play a positive role in protecting OA articular cartilage.^[30]^ The second is to improve or regulate metabolism through nutritional factors. Related Mendelian studies showed that the status of iron, copper, zinc, calcium, and selenium is associated with OA.^[31–33]^ Ruan Guangfeng’s research showed that dietary energy and fat intake have damage to knee OA, and starch and cellulose intake, calcium, magnesium, phosphorus, potassium, and iron elements protect knee OA, dietary factors on knee OA may be biomarker MMP 13, IL-8 and serum S100A8/S100A9 regulation of metabolism and inflammation of.^[34]^ By analyzing of minerals (calcium, magnesium, sodium, sulfur, B, aluminum, iron, manganese, copper, zinc, molybdenum, selenium), folic acid, and vitamin C, Louise et al The trace nutrients in dried fruit were 3 to 5 times the of fresh fruit.^[35]^ Third, it affects the composition of human intestinal flora. Cesarettin et al considered dried fruit (raisins, cranberries, jujube, and dried plum) to affect the composition of human intestinal flora in a potentially beneficial way (in terms of bifidobacterium, Clostria, Lactobacillus, Ruminoccaceae, Klebsiella, Prevotella), thus health benefits.^[26]^ The study showed that the pain of rat or human OA and inflammation and symptoms were reduced by administering probiotics, prebiotics, or drugs. These results suggest that dried fruit may affect the occurrence and progression of OA by affecting intestinal flora and its metabolites.^[36–38]^

This study also has some limitations: First, There is some heterogeneity in the MR analysis due to the use of the GWAS data’s ininability to explore any potential non-linear relationships or stratification effects that vary by age, health status, or gender; this may lead to the existence of heterogeneity; next, Although dried fruit intake has shown potential benefit in the prevention of OA, however, the specific type, quantity, quality and mechanism of action still need further research and clinical practice to determine; The aggregated GWAS data included only European ancestry or the ambiguous subjects, The conclusion may not be fully representative of other races, More studies should be conducted in the future to validate the applicability of these results to other ethnic populations.

## 5. Conclusion

In conclusion, the results of the MR analysis support that dried fruit intake may be causally related to reducing the risk of developing OA, indicating that dried fruit intake may reduce or delay the development of OA, which also provides evidence for dietary intervention to strengthen the prevention and management of OA.

## Acknowledgments

We extend our appreciation to theMRBase database (http://www.mrbase.org/) and IEU Open GWAS database (https://gwas.mrcieu.ac.uk/) for their invaluable contributions to our research.

## Author contributions

**Conceptualization:** Ruiming Liang, Weixing Zhong, Lixia Yuan.

**Data curation:** Ruiming Liang.

**Formal analysis:** Ruiming Liang, Weixing Zhong.

**Funding acquisition:** Lixia Yuan.

**Investigation:** Ruiming Liang, Weixing Zhong.

**Methodology:** Ruiming Liang.

**Project administration:** Lixia Yuan.

**Resources:** Ruiming Liang, Lixia Yuan.

**Software:** Weixing Zhong.

**Supervision:** Weixing Zhong, Lixia Yuan.

**Validation:** Weixing Zhong, Lixia Yuan.

**Visualization:** Ruiming Liang.

**Writing – original draft:** Ruiming Liang, Shuaidi Ze, Yuxiang Qiao.

**Writing – review & editing:** Ruiming Liang.
